# Risk and Severity of COVID-19 and ABO Blood Group in Transcatheter Aortic Valve Patients

**DOI:** 10.3390/jcm9113769

**Published:** 2020-11-22

**Authors:** Marion Kibler, Laurent Dietrich, Mohamad Kanso, Adrien Carmona, Benjamin Marchandot, Kensuke Matsushita, Antonin Trimaille, Cécile How-Choong, Albane Odier, Gabrielle Gennesseaux, Ophélie Schramm, Antje Reydel, Sébastien Hess, Chisato Sato, Sophie Caillard, Laurence Jesel, Olivier Morel, Patrick Ohlmann

**Affiliations:** 1 Division of Cardiovascular Medicine, Strasbourg University Hospital, 67000 Strasbourg, France; marion.kibler@chru-strasbourg.fr (M.K.); laurent_dietrich@yahoo.com (L.D.); mohamad.kanso@chru-strasbourg.fr (M.K.); adrien.carmona@chru-strasbourg.fr (A.C.); benjaminmarchandot@gmail.com (B.M.); matsuken_22@yahoo.co.jp (K.M.); antonin.trimaille@chru-strasbourg.fr (A.T.); howcec@hotmail.com (C.H.-C.); odieralbane@gmail.com (A.O.); gabrielle.gennesseaux@chru-strasbourg.fr (G.G.); ophelie.schramm@chru-strasbourg.fr (O.S.); anne-claire.reydel@chru-strasbourg.fr (A.R.); sebastien.hess@chru-strasbourg.fr (S.H.); okoge16@gmail.com (C.S.); laurence.jesel@chru-strasbourg.fr (L.J.); olivier.morel@chru-strasbourg.fr (O.M.); 2 INSERM (French National Institute of Health and Medical Research), UMR 1260, Regenerative Nanomedicine, FMTS, 67000 Strasbourg, France; 3 Division of Nephrology and Transplantation, Strasbourg University Hospital, 67000 Strasbourg, France; sophie.caillard@chru-strasbourg.fr

**Keywords:** ABO blood group, coronavirus disease 2019, transcatheter aortic valve replacement

## Abstract

While cardiovascular disease has been associated with an increased risk of coronavirus disease 2019 (COVID-19), no studies have described its clinical course in patients with aortic stenosis who had undergone transcatheter aortic valve replacement (TAVR). Numerous observational studies have reported an association between the A blood group and an increased susceptibility to SARS-CoV-2 infection. Our objective was to investigate the frequency and clinical course of COVID-19 in a large sample of patients who had undergone TAVR and to determine the associations of the ABO blood group with disease occurrence and outcomes. Patients who had undergone TAVR between 2010 and 2019 were included in this study and followed-up through the recent COVID-19 outbreak. The occurrence and severity (hospitalization and/or death) of COVID-19 and their associations with the ABO blood group served as the main outcome measures. Of the 1125 patients who had undergone TAVR, 403 (36%) died before 1 January 2020, and 20 (1.8%) were lost to follow-up. The study sample therefore consisted of 702 patients. Of them, we identified 22 cases (3.1%) with COVID-19. Fourteen patients (63.6%) were hospitalized or died of disease. Multivariable analysis identified the A blood group (vs. others) as the only independent predictor of COVID-19 in patients who had undergone TAVR (odds ratio (OR) = 6.32; 95% confidence interval (CI) = 2.11−18.92; *p* = 0.001). The A blood group (vs. others; OR = 8.27; 95% CI = 1.83−37.43, *p* = 0.006) and a history of cancer (OR = 4.99; 95% CI = 1.64−15.27, *p* = 0.005) were significantly and independently associated with disease severity (hospitalization and/or death). We conclude that patients who have undergone TAVR frequently have a number of cardiovascular comorbidities that may work to increase the risk of COVID-19. The subgroup with the A blood group was especially prone to developing the disease and showed unfavorable outcomes.

## 1. Introduction

At the end of 2019, a new zoonotic coronavirus (SARS-CoV-2)—responsible for coronavirus disease 2019 (COVID-19)—was reported in Wuhan, Hubei Province, China. The World Health Organization (WHO) declared the COVID-19 outbreak a global pandemic on 11 March 2020. SARS-CoV-2 spread rapidly in 166 other countries around the world, resulting in a global burden of 4,170,424 laboratory-confirmed cases and a death toll of 287,399 as of 14 May 2020 [1]. The Alsace region in eastern France has been significantly impacted, resulting in a rapid reshaping of in-hospital facilities. Several cardiology divisions have been converted into dedicated COVID-19 units, with cardiac care units being repurposed as intensive care units (ICUs) [2,3].

A history of cardiovascular (CV) disease is currently recognized as a risk factor for the occurrence and severity of COVID-19, especially in the elderly [4,5]. Previous studies have indicated that up to 40% of patients who required ICU admission for COVID-19 had preexisting congestive heart failure; further, the mortality rate from COVID-19 for patients with preexisting CV disease may be as high as 36% [6]. While there is ample literature to suggest a direct role for a history of heart disease in the susceptibility and severity of COVID-19, its clinical course in patients with valvular disease remains poorly investigated. With a growing number of patients with aortic stenosis being treated with transcatheter aortic valve replacement (TAVR), there is a strong need to investigate this interaction further.

Much of the recent focus in COVID-19 research has revolved around biological markers of disease susceptibility and/or severity. The ABO blood group has been shown to affect individual vulnerability to SARS-CoV [6], hepatitis B virus [7], Norwalk virus [8], and *Helicobacter pylori* infection [9]. Notably, observational studies have found an association between the A blood group and an increased susceptibility to SARS-CoV-2 infection [10,11,12].

The present study was undertaken to evaluate the clinical course of COVID-19 in patients with aortic stenosis who had undergone TAVR. We also examined whether the ABO blood group is associated with the susceptibility to and severity of COVID-19 in this clinical population, and whether this association is independent of potential confounders.

## 2. Materials and Methods

### 2.1. Study Setting and Patient Enrollment

This was a retrospective, observational investigation aimed at examining the occurrence and severity of COVID-19 in a large population of patients who had undergone TAVR for severe aortic stenosis between 2010 and 2019. The study was conducted in the Strasbourg University Hospital (Strasbourg, Alsace, eastern France). The general characteristics of the study patients—including demographics, medical history, echocardiography findings, and ABO blood group—were determined from their medical records and entered into an electronic file along with follow-up data. During the COVID-19 outbreak, all patients were contacted by phone to ascertain their health status, cardiovascular and COVID-19 symptoms, medication use, and outcomes. Patient-reported data collected through a standardized questionnaire were thoroughly cross-checked with official clinical records. The study was reviewed and approved by the Institutional Review Board at the Strasbourg University Hospital (CE-2020-69). Owing to the retrospective nature of the study, the need for informed consent was waived.

### 2.2. Definitions

In accordance with WHO technical guidance [13], patients were considered as confirmed cases of COVID-19 in the presence of positive reverse transcriptase-polymerase chain reaction (RT-PCR) testing of a nasopharyngeal swab specimen. Because RT-PCR can yield false-negative results, patients with typical symptoms and characteristic imaging findings on chest computed tomography (CT) were classified as confirmed cases [14]. Patients who were hospitalized for or died from COVID-19 were considered to have severe form of the disease. 

### 2.3. Statistical Analysis

Descriptive statistics are expressed as means ± standard deviations for continuous data or as counts (percentages) for categorical variables. Survival curves according to the ABO blood group were plotted by the Kaplan-Meier method (log-rank test) with right censoring at the time of last follow-up (8 May 2020). The time-to-event was calculated as the time elapsed from 1 January 2020 to the date of the index event (disease onset, hospitalization, or death). Logistic regression models were constructed to evaluate the unadjusted and covariate-adjusted odds ratios (ORs) and 95% confidence intervals (CIs) for the occurrence of COVID-19, COVID-19-related death, and severe COVID-19. All of the relevant parameters listed in Table 1 were entered as covariates in unadjusted models to test their univariate associations with the dependent variables. Variables adjusted for in the multivariable models were those showing univariate associations at a *p*-value < 0.20. We also included as covariates certain parameters that have previously been identified as risk factors for COVID-19 (i.e., age, male sex, cardiovascular comorbidities, cardiovascular risk factors, and chronic kidney disease). Statistical analyses were performed using SPSS, version 17.0 (IBM, Armonk, New York NY, USA). All tests were two-sided, and statistical significance was set as a *p*-value of <0.05.

## 3. Results

### 3.1. General Characteristics

**Figure 1:**
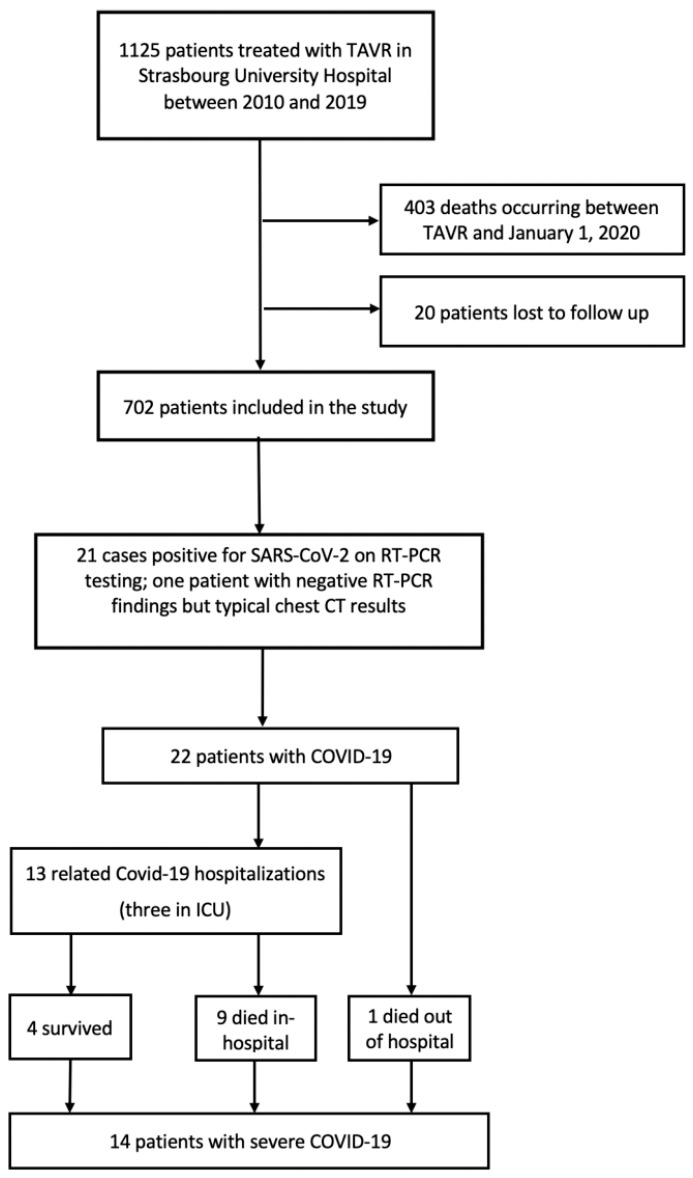
Flow chart of the study.

Between 2010 and 2019, a total of 1125 patients with aortic stenosis underwent TAVR in our hospital. We excluded 423 patients from the analysis due to death before 1 January 2020 (*n* = 403) or loss to follow-up (*n* = 20). Figure 1 depicts the flow of participants through the study. The general patient characteristics (*n* = 720; mean age: 82 ± 6.9 years; 44% men) are provided in Table 1. Common coexisting CV comorbidities included coronary artery disease (45.3%), atrial fibrillation (40.3%), congestive heart failure (35.9%), and peripheral arterial disease (27.2%). A positive history of cancer was present in 26.9% of cases, whereas chronic obstructive pulmonary disease and chronic kidney disease were identified in 11.7% and 16.5% of the study patients, respectively. At the time of interview, CV medications included angiotensin-converting enzyme (ACE) inhibitors/angiotensin II receptor blockers (48.9%), statins (50.2%), anticoagulants (45.4%), and aspirin (53.3%).

### 3.2. Occurrence and Presentation of COVID-19

Eighty-two patients (11.4%) had suspected COVID-19. Of them, 61 underwent RT-PCR testing and 21 chest CT. The diagnosis was confirmed in 22 cases (3.1%; 21 by RT-PCR and one by chest CT). 14 (63.6%) patients with confirmed COVID-19 were hospitalized or died of COVID-19. Common clinical symptoms at presentation included dyspnea (77.3%), fever (77.3%), and cough (72.7%). Myalgia, gastrointestinal manifestations, and anosmia/ageusia occurred in 40.9%, 27.3%, and 18.2% of participants, respectively.

### 3.3. COVID-19, Hospitalizations and Mortality

As of 1 January 2020, the all-cause and cardiovascular mortality rates in the study patients were 6.8% and 2.8%, respectively. Compared with patients without COVID-19, those with the disease had significantly higher all-cause mortality (5.6% vs. 45.5%, respectively; *p* < 0.001) and hospitalization (1.8% vs. 59.1%, respectively; *p* < 0.0001) rates (Table 2).

### 3.4. COVID-19 and ABO blood group

Patients with COVID-19 more frequently had the A blood group than those without (81.8% vs. 41.3%, respectively). Conversely, the O (18.2% vs. 46.5%, respectively), B (0% vs. 9.3%, respectively), and AB (0% vs. 2.9%, respectively) groups were underrepresented in patients with COVID-19. Subgroup analyses were subsequently performed according to the Rhesus (Rh) group. Interestingly, the A Rh+ blood type (68.2% vs. 29%, respectively)—but not the A Rh−type (13.6% vs. 12.4%, respectively)—was overrepresented in patients with COVID-19. The O Rh+ (9.1% vs. 22.4%, respectively), O Rh− (4.5% vs. 10.7%, respectively), B Rh+ (0% vs. 7.2%, respectively), B Rh− (0% vs. 2.1%, respectively), AB Rh+ (0% vs. 2.5%, respectively), and AB Rh− (0% vs. 1.1%, respectively) types were all underrepresented in patients with COVID-19 (Table 1). Additional analyses were also performed according to blood group A. Patients with the A blood group were more likely to develop COVID-19 compared to those with other blood types (6% vs. 1%, respectively; *p* < 0.0001). Additionally, patients with the A blood group more frequently experienced COVID-19-related death (3.4% vs. 0%, respectively; *p* < 0.0001) as well as the combined endpoint of COVID-19-related death or hospitalization (4% vs. 0.5%, respectively; *p* < 0.001; Tables S1 and S2).

### 3.5. Predictors of COVID-19

**Figure 2:**
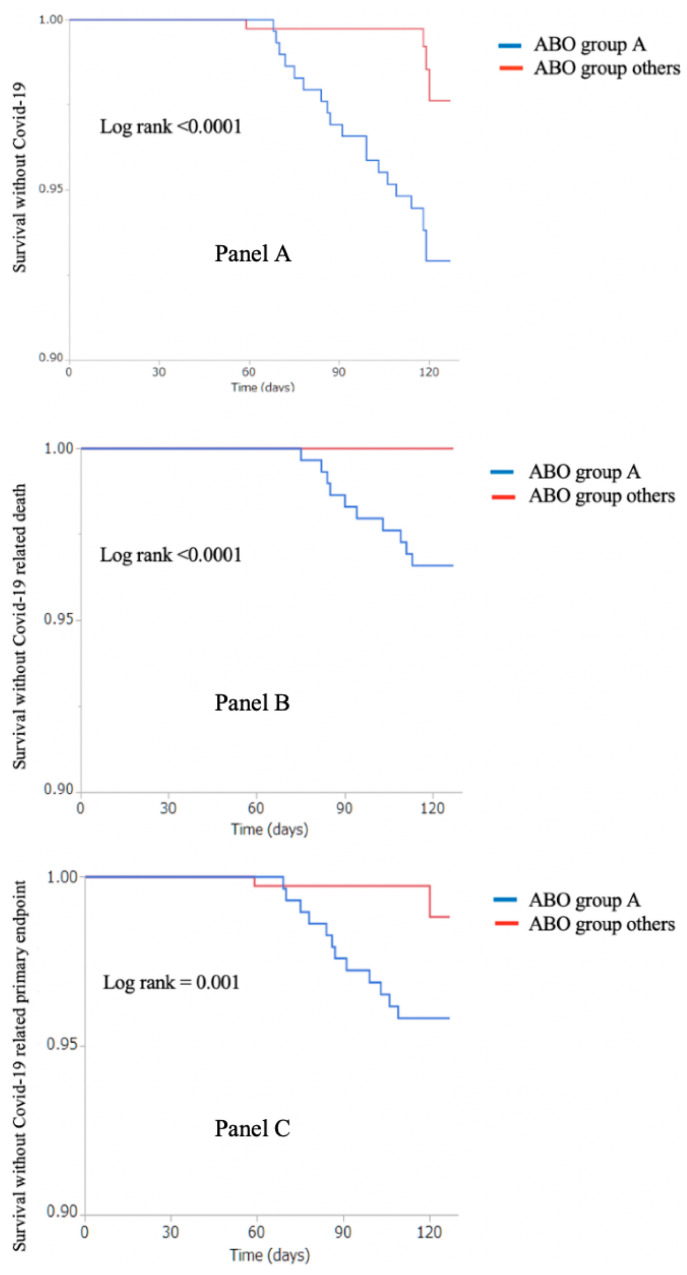
Kaplan–Meier plots of COVID-19-free survival (**panel A**), COVID-19-related mortality (**panel B**), and severe-COVID-19-free survival (**panel C**) according to the ABO blood group (group A versus other groups).

A history of cancer and blood type A were significant predictors of COVID-19 in the univariate analysis. Multivariable analysis identified the A blood group (vs. others) as the only independent predictor of COVID-19 in patients who had undergone TAVR (OR = 6.32; 95% CI = 2.11−18.92; *p* = 0.001; Table 3). Kaplan-Meier plots of COVID-19-free survival according to the blood group (A vs. others) are shown in Figure 2A.

### 3.6. Predictors of Severe COVID-19

Multivariable analysis (Table 4) revealed that blood group A (vs. others; OR = 8.27; 95% CI = 1.83−37.43, *p* = 0.006) and a history of cancer (OR = 4.99; 95% CI = 1.64−15.27, *p* = 0.005) were significantly and independently associated with COVID-19 severity (hospitalization and/or death). Kaplan-Meier plots of COVID-19-related mortality and severe-COVID-19-free survival are shown in Figure 2B,C, respectively.

## 4. Discussion

To our best knowledge, this is the first study to specifically investigate the impact of COVID-19 on patients who have undergone TAVR. There are two principal findings from our research. First, patients who had undergone TAVR were at high risk to contract COVID-19. Second, the A blood group was identified as a significant risk factor for both the occurrence and the severity of COVID-19.

### 4.1. Prevalence of COVID-19 

The prevalence of COVID-19 in our patients who had undergone TAVR was 3.13%, which is higher than that observed in the general French population (24/10,000 on 14 May 2020) [15].

Whether the increased COVID-19 rate was due to local characteristics of the outbreak in Alsace or to a higher susceptibility conferred by prior CV disease [16] needs further epidemiological study. Moreover, the mortality rate from COVID-19 was 45% in the current investigation. Age, frailty, and a significant burden of comorbidities are possible explanations for the high death toll [17]. Moreover, 63.6% of our patients had severe disease (hospitalization and/or death). Despite these findings, the mechanisms by which common risk factors for CV disease—including male sex, obesity or diabetes—confer susceptibility to COVID-19 remain unclear. Evidence regarding the association between aggressive disease and loss of ACE-2 function as a result of its proteolytic cleavage is emerging [18]. Under physiological conditions, ACE-2 counteracts the detrimental effects of angiotensin II [18], which might be overexpressed in patients with CV disease [19]. An imbalance of ACE-2 and ACE-1 activity at sites of endothelial injury may promote angiotensin II accumulation [20], which can further exacerbate tissue injury and lead to microvascular thrombotic disease [21,22]. Although our study does not address the role of ACE-2 in the susceptibility to COVID-19 among patients who had undergone TAVR, its involvement is certainly plausible.

### 4.2. ABO Blood Group and COVID-19

In the current study, patients who had undergone TAVR and had the A blood group were more prone to developing COVID-19 and were more likely to experience unfavorable outcomes.

In a study conducted in 2173 Chinese patients, Zhao et al. [23] showed for the first time that the A blood group was associated with an increased susceptibility to COVID-19 while the O group seemed less vulnerable. They also found higher death rates in patients with the A group. A more recent report from the Central Hospital of Wuhan confirmed the increased risk conferred by the A group and the reduced disease susceptibility associated with the O group [11]. Another study connecting the A blood group with an increased risk of contracting COVID-19 analyzed 1599 individuals who underwent SARS-CoV-2 testing in the United States [24]. However, no relation with in-hospital mortality was found. The authors carried these observations a step further with Rh antigen testing and found that its expression could modulate the association of the ABO blood group with disease susceptibility. Similar findings were noticed in our study, albeit limited to the A group. If a subject had the A group and was also Rh+, the patient would have a substantially higher risk of COVID-19, but this was not the case for Rh− individuals.

Growing evidence indicates that the A blood group is associated with an increased susceptibility to and severity of COVID-19. The mechanisms beyond this association are unknown, but several hypotheses might be raised. It is possible that anti-A antibodies could lead to a decreased interaction of SARS-CoV-2 with its cellular receptor ACE-2 [25]. Interestingly, the A blood group has also been related with an increased risk of CV disease [26]. Numerous biological pathways have been proposed to account for the association between the A blood group and atherothrombosis, including an increased production of soluble intercellular adhesion molecules [27] and/or von Willebrand factor (vWF) [28]. Other authors have emphasized the significance of vWF cleavage in subjects with the O blood group [29], an event which may reduce thrombotic risk in SARS-CoV-2-infected individuals [12]. Recently, a molecular genetic analysis of case/control data identified two loci (3p21.31 and 9q34.2) as significantly associated with severe COVID-19. Interestingly, the ABO gene resides on chromosome 9 at the band 9q34.2. Further, this study reported an increased risk of severe COVID-19 in patients with the A blood group (OR: 1.45) whereas the O blood group had a protective effect (OR: 0.65) [30].

### 4.3. Limitations

Several caveats of our investigation need to be considered. First, our study employed a retrospective design and the number of observed events (deaths and/or hospitalizations) was limited. As such, the presence of residual confounding may pose limitations in our ability to generalize our conclusions. Second, our research has an exploratory nature and we cannot rule out the presence of chance findings resulting from multiple comparisons. Another caveat is that the sex distribution of participants varied across the ABO blood groups. Specifically, men were underrepresented in the A blood group, potentially posing limitations in our ability to fully explore the impact of this variable on COVID-19 severity. This study was not designed to evaluate the clinical management of aortic stenosis during the ongoing COVID-19 pandemic. However, recent research suggests that patients with aortic stenosis should not currently undergo TAVR unless in the presence of severe disease [31,32,33]. Such an approach may serve to reduce potential exposure to SARS-CoV-2 during hospitalization. Finally, TAVR is not per se a predisposing factor for COVID-19. Several other surgical or interventional procedures may be associated with an increased risk of COVID-19 if they are conducted in patients with similar cardiovascular risk profiles and a comparable burden of comorbidities.

## 5. Conclusions

Patients who have undergone TAVR frequently have a number of cardiovascular comorbidities that may work to increase the risk of COVID-19. The subgroup with the A blood group was especially prone to develop the disease and showed unfavorable outcomes. Our results add to the growing body of literature indicating that the ABO blood group may be a useful laboratory parameter that should be taken into account for risk stratification during the clinical work-up of patients with COVID-19.

## Figures and Tables

**Table 1:** General characteristics of patients who had undergone transcatheter aortic valve replacement according to the presence or absence of COVID-19.

Clinical Characteristics	Entire Cohort (*n* = 702)	COVID-19 (*n* = 22)	No COVID-19 (*n* = 680)	*p* Value
Age, years	82 ± 6.9	82 ± 8.4	82 ± 6.9	0.961
Male sex–*n* (%)	313 (44)	7 (31.8)	306 (45)	0.220
STS score–%	5.9 ± 4.9	5.5 ± 2.4	5.9 ± 5.0	0.757
**Cardiovascular risk factors–*n* (%)**				
Current smoking	26 (3.7)	1 (4.5)	25 (3.7)	0.832
Hypertension	587 (83.6)	18 (81.8)	569 (83.7)	0.817
Obesity (Body mass index > 30 kg/m^2^)	183 (26.1)	6 (27.3)	177 (26.1)	0.899
Dyslipidemia	428 (61)	12 (54.5)	416 (61.2)	0.530
Diabetes	213 (30.3)	6 (27.3)	207 (30.4)	0.750
**Comorbidities–*n* (%)**				
Coronary artery disease	318 (45.3)	12 (54.5)	306 (45.0)	0.376
Congestive heart failure	252 (35.9)	6 (27.3)	246 (36.5)	0.392
Stroke	98 (14)	3 (13.6)	95 (14.0)	0.964
Atrial fibrillation	283 (40.3)	6 (27.3)	277 (40.7)	0.205
Peripheral arterial disease	191 (27.2)	5 (22.7)	186 (27.4)	0.631
COPD	82 (11.7)	3 (13.6)	79 (11.6)	0.740
Prior cancer	189 (26.9)	10 (45.5)	179 (26.3)	0.053
CKD (Creatinine levels > 130 μmol/L)	115 (16.5)	4 (18.2)	111 (16.4)	0.824
LVEF after TAVR–%	56 ± 11	56 ± 12	56 ± 11	0.902
**Treatment at time of follow up**–*n* (%)				
Aspirin	365 (53.3)	13 (59.1)	352 (53.1)	0.579
**P2Y12 inhibitors**				
VKA	144 (21.0)	4 (18.2)	140 (21.1)	0.740
DOAC	175 (25.5)	6 (27.3)	169 (25.5)	0.850
ACE-i/ARB	335 (48.9)	12 (54.5)	323 (48.7)	0.591
Statins	344 (50.2)	9 (40.9)	335 (50.5)	0.375
Amiodarone	101 (14.7)	2 (9.1)	99 (14.9)	0.447
**ABO blood type**–*n* (%)				
A	299 (42.6)	18 (81.8)	281 (41.3)	0.002
B	63 (9)	0 (0)	63 (9.3)	
AB	20 (2.9)	0 (0)	20 (2.9)	
O	320 (45.6)	4 (18.2)	316 (46.5)	
Rhesus positive (Rh+)–*n* (%)	352 (58.6)	11 (68.8)	341 (58.3)	0.402
**Blood type**–no. (%)				
A Rh-	87 (12.4)	3 (13.6)	84 (12.4)	0.027
A Rh+	212 (30.2)	15 (68.2)	197 (29.0)	
AB Rh-	8 (1.1)	0 (0)	8 (1.2)	
AB Rh+	17 (2.4)	0 (0)	17 (2.5)	
B Rh-	14 (2.0)	0 (0)	14 (2.1)	
B Rh+	49 (7.0)	0 (0)	49 (7.2)	
O Rh-	74 (10.5)	1 (4.5)	73 (10.7)	
O Rh+	154 (21.9)	2 (9.1)	152 (22.4)	
Missing	87 (12.4)	1 (4.5)	86 (12.6)	

Data are given as means ± standard deviations or counts (percentages). Abbreviations: ACE-i: Angiotensin Converting Enzyme inhibitor; ARB: Angiotensin Receptor blocker; CKD: Chronic Kidney Disease (creatinine > 130 μmol/L); COPD: Chronic Obstructive Pulmonary Disease; COVID-19: Coronavirus Disease 2019; DOAC: direct oral anticoagulant; LVEF: Left Ventricular Ejection Fraction; STS score: Society of Thoracic Surgeons score; TAVR: Transcatheter Aortic Valve Replacement; VKA: vitamin K antagonist.

**Table 2:** Clinical outcomes of patients who had undergone transcatheter aortic valve replacement according to the presence or absence of COVID-19.

	Entire Cohort (*n* = 702)	COVID-19 (*n* = 22)	No COVID-19 (*n* = 680)	*p* Value
**Hospitalization–*n* (%)**	25 (3.6)	13 (59.1)	12 (1.8)	<0.0001
Conventional unit	22 (3.2)	10 (45.5)	12 (1.8)	<0.0001
Intensive care unit	3 (0.44)	3 (13.6)	0 (0)	<0.0001
**Mortality from January 1, 2020–*n* (%)**				
All-cause mortality	48 (6.8)	10 (45.5)	38 (5.6)	<0.0001
Cardiovascular mortality	20 (2.8)	0 (0)	20 (2.8)	0.414
COVID-19 mortality	10 (1.5)	10 (45.5)	0 (0)	<0.0001
COVID-19 severity–*n* (%)				
COVID-19 related hospitalization or death	14 (2.0)	14 (63.6)	0 (0)	<0.0001

Abbreviations: COVID-19: Coronavirus Disease 2019.

**Table 3:** Factors associated with the occurrence of COVID-19 in patients who had undergone transcatheter aortic valve replacement.

	Univariate Analysis			Multivariate Analysis		
	OR	95% CI	*p* Value	OR	95% CI	*p* Value
Age	0.99	0.94–1.06	0.610			
Male sex	0.57	0.23–1.42	0.226			
Diabetes	0.86	0.33–2.22	0.751			
Obesity	0.89	0.41–2.76	0.899			
Hypertension	0.89	0.29–2.64	0.817			
Dyslipidemia	0.76	0.32–1.79	0.531			
Current smoking	0.83	0.16–9.65	0.832			
Atrial fibrillation	0.55	0.21–1.41	0.212			
Peripheral artery disease	0.78	0.28–2.15	0.632			
CKD (Creatinine levels > 130 umol/L)	1.13	0.38–3.41	0.703			
Prior cancer	2.33	0.99–5.49	0.053	2.28	0.96–5.43	0.062
ACE-i/ARBs	1.26	0.54–2.96	0.591			
P2Y12 inhibitors	0.70	0.09–5.37	0.736			
Aspirin	1.28	0.54–3.03	0.580			
Statins	0.68	0.29–1.61	0.377			
A blood group	6.29	2.14–19.08	0.001	6.32	2.11–18.92	0.001

Abbreviations: ACEi, angiotensin converting enzyme inhibitors; ARB, angiotensin receptor blockers; CI, confidence interval; CKD, chronic kidney disease; COVID-19, coronavirus disease 2019; OR, odds ratio.

**Table 4:** Factors associated with severe COVID-19 in patients who had undergone transcatheter aortic valve replacement.

	Univariate Analysis			Multivariate Analysis		
	OR	95% CI	*p* Value	OR	95% CI	*p* Value
Age	0.98	0.92–1.05	0.649			
Sex (male)	0.68	0.23–2.07	0.502			
Diabetes	1.74	0.59–5.09	0.309			
Obesity	0.77	0.21–2.78	0.688			
Hypertension	0.71	0.19–2.59	0.608			
Dyslipidemia	0.85	0.29–2.48	0.767			
Atrial fibrillation	0.57	0.18–1.69	0.371			
Peripheral artery disease	1.07	0.33–3.47	0.908			
CKD (Cr > 130 umol/L)	2.07	0.64–6.71	0.226			
Coronary artery disease	1.63	0.56–4.74	0.373			
Heart failure	0.71	0.22–2.29	0.566			
COPD	1.26	0.28–5.75	0.761			
Stroke	1.03	0.23–4.66	0.972			
Prior cancer	5.08	1.68–15.34	0.004	4.99	1.64–15.27	0.005
A blood group	8.38	1.86–37.74	0.006	8.27	1.83–37.43	0.006
O blood group	0.19	0.04–0.87	0.033			
Aspirin	0.87	0.30–2.52	0.804			
ACE-i/ARB	1.05	0.36–3.01	0.934			
Statins	0.74	0.25–2.15	0.579			

Abbreviations: ACE-i, angiotensin converting enzyme inhibitors; ARB, angiotensin receptor blockers; CI, confidence interval; CKD, chronic kidney disease; COPD, chronic obstructive pulmonary disease; COVID-19, coronavirus disease 2019.

## Supplementary Materials

The following are available online at https://www.mdpi.com/2077-0383/9/11/3769/s1; Table S1. Clinical outcomes of patients who had undergone transcatheter aortic valve replacement according to the A blood group, Table S2. General characteristics of patients who had undergone transcatheter aortic valve replacement according to the presence or A blood group vs. other groups.
