# Supplementary material for: Risk and Severity of COVID-19 and ABO Blood Group in Transcatheter Aortic Valve Patients
Source: J Clin Med. 2020 Nov 22;9(11):3769. doi: 10.3390/jcm9113769 (PMC7700222; doi:10.3390/jcm9113769)
Supplement: Supplementary file 1 [file jcm-09-03769-s001.pdf]

Table S1. Clinical outcomes of patients who had undergone transcatheter aortic valve replacement according to A blood group.

Variable	Entire Cohort (n = 702)	A Blood Group (n = 299)	Others Blood Groups (n = 403)	p Value
**Confirmed COVID-19**	22 (3.1)	18 (6)	4 (1)	<0.0001
**Mortality**				
COVID-19 mortality	10 (1.5)	10 (3.4)	0 (0)	<0.0001
**COVID-19 severity–*n* (%)**				
COVID-19 related hospitalization or death	14 (2.0)	12 (4.0)	2 (0.5)	0.001

Abbreviations: COVID-19: Coronavirus disease 2019.

Table S2. General characteristics of patients who had undergone transcatheter aortic valve replacement according to the presence or A blood group versus other groups.

Variable	Entire Cohort (n = 702)	A Blood Group (n = 299)	Other Blood Groups (n = 403)	p Value
Age, years	82.6 ± 6.9	82.4 ± 7.1	82.8 ± 6.9	0.513
Male sex–*n* (%)	313 (44.6)	116 (38.8)	197 (48.9)	0.008
STS score–%	5.9 ± 5.0	6.0 ± 5.1	5.8 ± 4.9	0.645
**Cardiovascular risk factors–*n* (%)**				
Current smoking	26 (3.7)	8 (2.7)	18 (4.5)	0.214
Hypertension	587 (83.6)	253 (84.6)	334 (82.9)	0.539
BMI (kg/m^2)	27.3 ± 5.8	27.3 ± 5.8	27.2 ± 5.6	0.892
Dyslipidemia	428 (61.0)	186 (62.2)	242 (56.5)	0.562
Diabetes	213 (30.3)	97 (32.4)	116 (28.8)	0.297
**Comorbidities–*n* (%)**				
Coronary artery disease	318 (45.3)	146 (48.8)	172 (42.7)	0.106
Congestive heart failure	252 (35.9)	107 (35.8)	145 (36.0)	0.958
Stroke	98 (14.0)	40 (13.4)	58 (14.4)	0.701
Atrial fibrillation	283 (40.3)	121 (40.5)	162 (40.2)	0.943
COPD	82 (11.7)	36 (12.1)	46 (11.4)	0.786
Prior cancer	187 (26.6)	82 (27.4)	105 (26.1)	0.685
CKD (Creatinine levels > 130 mol/L)	102 (14.6)	50 (16.8)	52 (12.9)	0.150
Peripheral arterial disease	191 (27.2)	84 (28.1)	107 (26.6)	0.650
**Blood Type–*n* (%)**				
A B AB O	299 (42.6)	299 (100)	0 (0)	<0.0001
	63 (9.0)	0 (0)	63 (15.6)	
	20 (2.8)	0 (0)	20 (5.0)	
	320 (45.6)	0 (0)	320 (79.4)	
**Treatment at the time of follow up–*n* (%)**				
Aspirin	365 (53.3)	163 (54.9)	202 (52.1)	0.463
P2Y12 inhibitors	44 (6.3)	19 (6.4)	25 (6.2)	0.935
VKA	144 (21.0)	58 (19.5)	86 (22.2)	0.401
DOAC	175 (25.5)	78 (26.3)	97 (25.0)	0.707
ACE-i/ARB	335 (48.9)	152 (51.2)	183 (47.2)	0.298
Statins	344 (50.2)	156 (52.5)	188 (48.5)	0.291

Data are given as means ± standard deviations or counts (percentages). Abbreviations: ACE-i, angiotensin converting enzyme inhibitors; ARB, angiotensin receptor blockers; BMI: Body mass index; CKD, chronic kidney disease; COPD, chronic obstructive pulmonary disease; COVID-19, coronavirus disease 2019; DOAC, direct oral anticoagulants; STS, Society of Thoracic Surgeons; VKA, vitamin K antagonist.
